# A Deep Learning Approach for Meter-Scale Air Quality Estimation in Urban Environments Using Very High-Spatial-Resolution Satellite Imagery

**DOI:** 10.3390/atmos13050696

**Published:** 2022-04-27

**Authors:** Meytar Sorek-Hamer, Michael von Pohle, Adwait Sahasrabhojanee, Ata Akbari Asanjan, Emily Deardorff, Esra Suel, Violet Lingenfelter, Kamalika Das, Nikunj Oza, Majid Ezzati, Michael Brauer

**Affiliations:** a Universities Space Research Association (USRA), Mountain View, CA; b NASA Ames Research Center, Mountain View, CA; c Imperial College, London, UK; d University of British Columbia, SPPH, Vancouver, Canada

**Keywords:** Air quality, Remote Sensing, Urban environment, Deep learning, Satellite Imagery

## Abstract

High-spatial-resolution air quality (AQ) mapping is important for identifying pollution sources to facilitate local action. Some of the most populated cities in the world are not equipped with the infrastructure required to monitor AQ levels on the ground and must rely on other sources, like satellite derived estimates, to monitor AQ. Current satellite-data-based models provide AQ mapping on a kilometer scale at best. In this study we focus on producing hundred-meter-scale AQ maps for urban environments in developed cities. We examined the feasibility of an image-based object-detection analysis approach using very high-spatial-resolution (2.5 m) commercial satellite imagery. We fed the satellite imagery to a deep neural network (DNN) to learn the association between visual urban features and air pollutants. The developed model, which solely uses satellite imagery, was tested and evaluated using both ground monitoring observations and land-use regression modeled PM_2.5_ and NO_2_ concentrations over London, Vancouver (BC), Los Angeles, and New York City. The results demonstrate a low error with a total RMSE < 2 µg/m^3^ and highlight the contribution of specific urban features, such as green areas and roads, to continuous hundred-meter-scale AQ estimation. This approach offers promise for scaling to global applications in developed and developing urban environments. Further analysis on domain transferability will enable application of a parsimonious model based merely on satellite images to create hundred-meter-scale AQ maps in developing cities, where current and historical ground data is limited.

## 1 Introduction

Air quality in urban environments results from a complex interaction between environmental conditions and natural and anthropogenic sources. The air we breathe has a strong impact on our health and life expectancy (Pope *et al* 2019, Brauer 2017). Poor air quality has been consistently ranked among the top risk factors for death and disability worldwide. In 2017, air pollution was the fifth-highest mortality risk factor globally and was associated with about 4.9 million deaths (HEI 2019). Fine particulate matter PM_2.5_ and Nitrogen Dioxide NO_2_, are common urban air pollutants (Wang *et al* 2020, Kotchenruther 2016). While NO_2_ is especially important in urban areas and a marker for traffic-related air pollution, mainly affected by local sources (e.g. transportation), PM_2.5_ is most relevant for health impacts and has a regional effect with numerous contributors (Chi Nguyen *et al* 2018, Karagulian *et al* 2015).

Many epidemiological studies examining the health effects of air pollution have measures of air pollutant concentrations collected from sparse networks of stationary ground monitors as their main exposure metric. Continuous, high-spatial-resolution air quality (AQ) estimates will help with source identification, facilitate local awareness, improve the accuracy and specificity of health effects studies, and be useful for tracking impacts of air quality management.

Over the past several decades, different models have been used to estimate air quality, with improved spatial coverage and reduced bias. These include dispersion modeling (Isakov *et al* 2019), regression kriging (van Zoest *et al* 2020), land-use regression (LUR) (Jin *et al* 2019, de Hoogh *et al* 2016, Bertazzon *et al* 2015, Beelen *et al* 2013, Ross *et al* 2007), and satellite-based models (van Donkelaar *et al* 2016, Kloog *et al* 2015, Engel-Cox *et al* 2004). The satellite-based models use retrieved variables, like Aerosol Optical Depth (AOD), as explanatory variables. Satellite-based estimates are increasingly being used to determine continuous exposure metrics in health studies at coarse resolutions (1 km–10 km) and with limitations related to column-surface calibration. Assessments of long-term air pollution exposure in environmental health studies have commonly employed LUR or chemical transport modeling (CTM) techniques. While CTM requires local emissions data, LUR is a commonly used algorithm for urban regions that requires substantial local (measured) data such as traffic, meteorology, and spatially disaggregated population data. LUR requires gathering many datasets on a specific location and therefore is usually performed on an annual basis (de Hoogh *et al* 2016, Beelen *et al* 2013). Due to large input data requirements of LUR models, coverage cannot easily be scaled up. As a result, we currently lack high-spatial-resolution estimates of pollutant levels in many cities around the world especially in low- and middle-income countries.

LUR modeling uses multiple regression equations to describe the relationship between sample locations and environmental variables. Resulting models can predict pollution concentrations at unmeasured locations, usually with relatively high spatial resolution. In addition, satellite-based retrieved data (e.g. AOD, temperature) have been used for estimating concentrations of air pollutants mostly combined with additional explanatory variables (Hammer *et al* 2020, Sorek-Hamer *et al* 2020, van Donkelaar *et al* 2010, Franklin *et al* 2018). The great allure of satellite data is its global coverage. These models perform well where historical ground monitoring data and ancillary data are available and calibration is possible, which facilitates production of continuous air pollutant concentration maps at a relatively coarse spatial resolution (i.e., on the kilometer scale).

The spatial resolution of available satellite imagery has increased significantly due to the existence of commercial satellites that obtain daily images of the globe at a meter-scale resolution. Current developments in deep learning methods have been recently introduced to the field to improve capabilities of estimating socioeconomic and environmental factors using satellite imagery and not only satellite-borne retrieved variables, as AOD (Suel *et al* 2019, Maharana and Nsoesie 2018, Jean *et al* 2016), including air quality (Yan et al. 2020, Yan et al. 2021). Visual features in high-spatial-resolution satellite imagery contain significant information relevant to urban air quality. This will allow the next generation of air quality models to estimate hundred-meter-scale air quality in urban environments potentially without requiring any locally collected ground data.

This study demonstrates a novel deep learning approach based on very high-spatial-resolution satellite imagery (2.5 m) to estimate PM_2.5_ and NO_2_ annual mean concentrations in urban environments. The analysis was conducted on Greater London, UK; Vancouver, Canada; and Los Angeles (LA), USA and evaluated on an unseen city—New York City (NYC), USA in 2010 (datasets and methodology are detailed in the ‘Data and Methods’ section and in the Appendix). Results from the multi-location model are validated with ground measurements and with 2010 LUR models (which serve as our target data). Furthermore, spatial error analysis and model interpretability are discussed.

## 2 Data and Methods

### 2.1 Data

#### Satellite Imagery

We used Maxar (formerly DigitalGlobe) WorldView2 (WV2) images available over Greater London, Vancouver, LA, and NYC for the year 2010. WV2 was launched on October 8, 2009, as part of the Maxar satellite constellation. It is a Sun-synchronous satellite with a 10:30 a.m. descending node overpass, located 770 km from Earth. Images are produced in 8 spectral bands in the VIS–NIR range (400 nm–1040 nm) with a spatial resolution of 50 cm (for the panchromatic imagery) and 2.5 m (for the multi-spectral imagery) (DigitalGlobe 2016). Access to the Maxar imagery archive was completed under the ‘NextView’ license framework. Over the study period we obtained and pre-processed raw imagery over all study areas, including radiometric correction and cloud cleaning, resulting in a total of 717 non-overlapping images that were used to construct 612,248 image patches from the 2.5m spatial resolution satellite imagery (Table 1).

#### LUR data

We obtained annual mean LUR-modeled PM_2.5_ and NO_2_ concentrations (for 2010) with 100 m and 200 m spatial resolution, respectively (NYCCAS 2018, de Hoogh *et al* 2016, Beelen *et al* 2013, Bechle *et al* 2015, Henderson *et al* 2007). These continuous surfaces served as the target data for training, testing, and validating the model. For further evaluation we calculated annual values for each ground monitoring site for all study regions. An example list of variables included in the LUR can be found in the SI Section B. with more details in de Hoogh et al. 2016.

To efficiently use the meter-scale satellite images with the ability to have a target dataset for testing and validating the developed model, we looked for a reliable continuous surface that is commonly used. Since a ground monitoring network isn’t a continuous mapping surface, it does not provide sufficient numbers of labeled data to train deep learning models. As an alternative, we proposed to use Land Use Regression model (LUR) outputs, which are developed based on real data, e.g. air quality monitors, traffic density, distance from roads and rails, population density among others. LURs require much effort in obtaining the data and are limited for regions that have that data available. It has been used in environmental health and urban planning studies as a valid exposure metrics (Gulliver et al. 2018, Hoek 2017, Xie et al, 2011). The correlations between 2010 LUR PM2.5/NO2 models and ground monitoring concentrations, from available ground monitoring sites at the study metropolitan areas range from 0.262 in London to 0.44 in New York for PM2.5 and 0.1 in London to 0.78 in Vancouver for NO_2_. These calculated correlations are in agreement with published results in Europe between LUR and ground monitoring sites (de Hoogh et al. 2016).

In this study, we are mapping AQ in the 100-200m scale, defined by the target data (i.e., LUR) resolution. This finer spatial resolution expresses human’s exposure. It addresses local AQ distribution, AQ spatial variation that cannot be captured in lower spatial resolutions. These high spatial resolutions are key for detecting pollution sources and are a proxy for human activity and urban planning. We use information from 2.5 m spatial-resolution satellite imagery to understand the relationship between PM2.5/NO2 and surface reflectance (in a broader look). The advantage of using very high-spatial-resolution satellite images is the ability to capture urban features that are known to influence for pollution levels (e.g., roads, buildings, trees) that are often used as inputs to LUR models but not visible at lower resolutions. This approach helps our proposed model to account for more than a single pixel (2.5x2.5 m^2^ area) but understand the urban composition causing that pollution level.

This study introduces an opportunity to use meter-scale images which are globally available to develop a more parsimonious model for estimating AQ. This approach can then be applicable in developed and developing urban environments that lack the data required for developing LUR models.

#### Ground monitoring data

Ground monitoring data of PM_2.5_ and NO_2_ concentrations in [ug/m^3^] were obtained for 2010 at all study areas and annual concentrations were calculated. For London, daily ground monitoring data was used to calculate annual means of measured pollution over ground stations that had at least one measured value every month. For the other cities, annual means of measured data were used for the ground monitoring sites that had collected data for over 50% of the year. Only sites that had co-located model-estimated values within a distance less than 500 m were included. We used a total of 33 (31) ground PM_2.5_ (NO2) sites, for evaluation, with 11 (7), 8 (12), 8 (10), and 6 (2) sites in London, Vancouver, LA, and NYC, respectively. Data was obtained from the following sources: London (Lang et al. 2019, Kings College, 2022), Vancouver (Government of Canada, 2022), LA (US EPA 2022), and NYC (NYCCAS, 2018, US EPA 2022).

### 2.2 Methodology

In this study, we used over 700 Maxar satellite images to train, validate, and test our air quality estimation models. The imagery has a spatial resolution of 2.5 m, covered 4 cities, and came from the year 2010 (see details in Table 1).

In the first stage, the model was trained and validated on combined data from three urban environments: London, Vancouver, and LA. This multi-location model was then tested to predict PM2.5 and NO2 concentrations in the same three locations, on a subset of data unseen by the model. In the second stage, we validated the same trained model on NYC: an urban region completely unseen by the model.

The same two-stage process was used to produce separate models for each air pollutant, and both models were trained and evaluated with LUR estimates as their target data (more details in ‘Data and Methods’ section). Root Mean Squared Error (RMSE), residuals (defined as the difference between the predicted values and the target data), and Normalized RMSE (NRMSE, defined as RMSE divided by the difference between the 25th and 75th percentiles of the target PM2.5/NO2 concentrations) were calculated to quantify model prediction errors. In addition, we compare our results to available ground monitoring stations' annual concentrations for PM2.5 and NO2.

#### Model Architecture

**Fig. 1:**
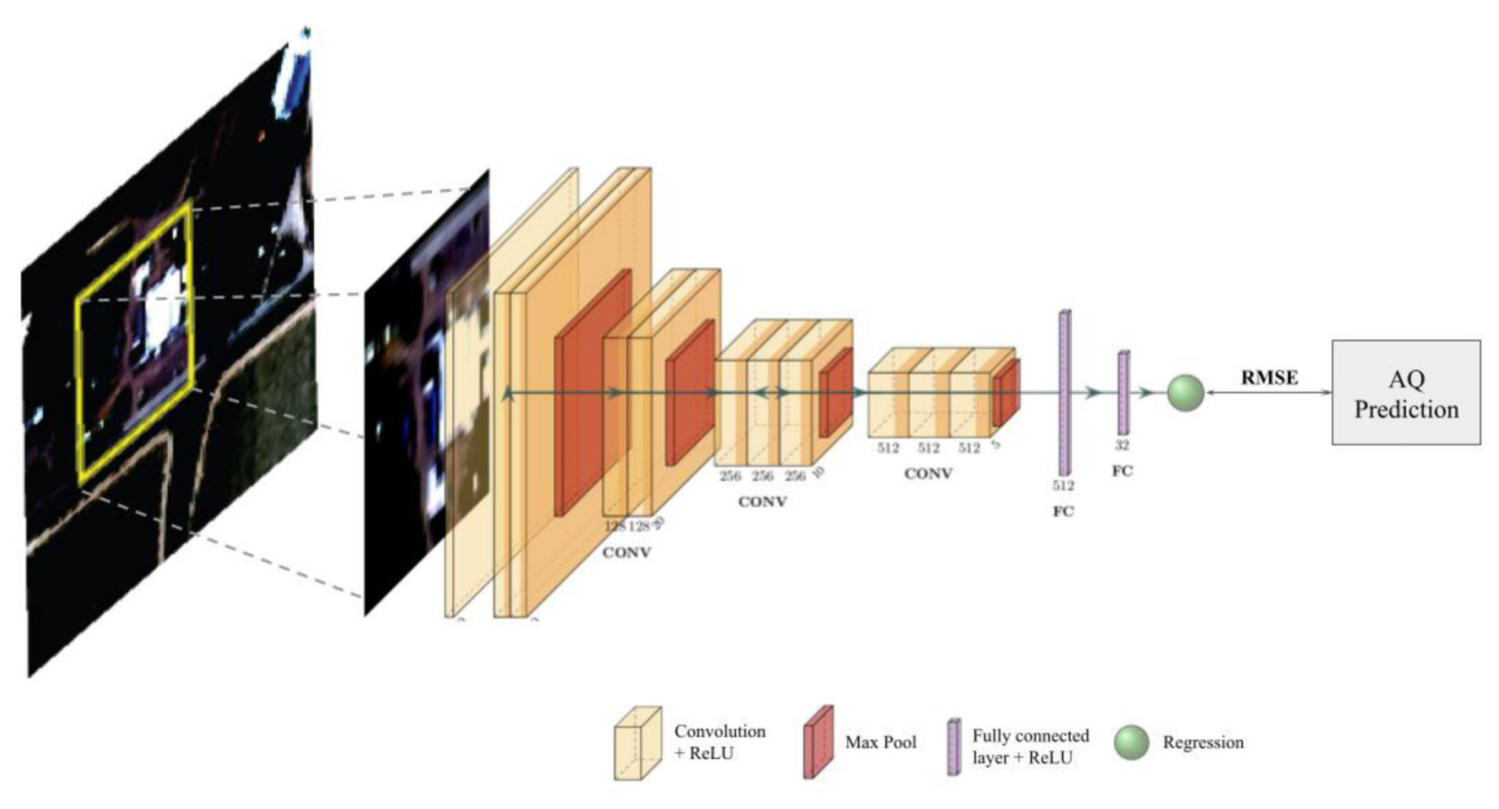
Methodology Flowchart Single satellite imagery patches are input to the CNN 40x40 pixel (100mx100m) for PM2.5 and 80x80 pixels (200mx200m) for NO2, each patch corresponds to one target value from a continuous LUR surface. The satellite images are applied to a convolutional neural network (CNN) which consists of stacked layers of convolution and nonlinear activation functions (ReLU) (yellow blocks) followed by Max Pooling layers (Red blocks) to spatially down-sample the features at the end of each convolutional block. Lastly, the convolutional features are flattened and fed into Fully connected layers (showed in purple) and regressed to the LUR training data to predict continuous PM2.5/NO2 estimates. Root Mean Squared Error (RMSE) was used to measure the error and evaluate the model. (Further details: SI Section A.).

We used a deep learning approach to develop a new model for estimating PM_2.5_ and NO_2_ concentrations. We modified a VGG16 model (developed by Simonyan et al. (2014)) which is a deep convolutional neural network (CNN) originally tasked to classify objects in the ImageNet dataset. CNNs are a type of neural networks which are popular for their effective and efficient performances in dependent data structures such as time-series and images (LeCun & Bengio, 1995). CNNs consist of a stack of convolutional layers that will understand data patterns using internal data dependency. For instance, in images, each pixel is dependent on its neighboring pixels and the image patterns are linked to not just a pixel but a collection of pixels in a neighborhood. Hence, CNNs are capable of learning spatial features, such as roads, buildings, trees, etc. in urban reflectance images from satellite data. The aim of this study is to train a CNN that efficiently and effectively learns the urban features and finds their relations to the PM_2.5_ and NO_2_ pollution level. To adapt the VGG16 model to our objective, we changed the original architecture from a classification model to a regression model by removing the final softmax activation function (resulting in a neuron with linear output). This enabled us to estimate continuous PM_2.5_ and NO_2_ concentration levels. We also removed some of the convolutional layers to account for the difference in size of our input imagery. The new model is trained to learn urban reflectance patterns and estimate their corresponding PM_2.5_ and NO_2_ values (Fig. 1, Fig. S1). This approach was possible because of the computational resources of the NASA Ames Research Center High-End Computing Capability GPU nodes. The proposed model is setup to take Maxar images with a spatial resolution of 2.5 meters and output AQ maps with 100-200 meters scale. The model will take a high-spatial-resolution image (meter-scale) as input and output a corresponding value with 100-200 meters scale. Thus, we won’t see the local pixelated behavior and the model output will have the same resolution/detail level as the LUR target data.

#### Data Preparation

To prepare our data for model training, we aligned our satellite imagery from the study areas with the LUR models and partitioned the imagery into patches that matched the grid resolution of the LUR-modeled data. This resulted in 100 m (200 m) 40 x 40 (80 x 80) pixel patches for the PM_2.5_ (NO_2_) data. Some locations in our study area have multiple overlapping satellite images, which produces multiple patches for the same target data point. We randomly selected one of these patches for each point so that our dataset has only one patch corresponding to each target data point. Our imagery dataset has a disproportionate number of image patches for some cities over others, with LA having over 3 times as many patches as London. To prevent the model from favoring the image distribution of one city, we balanced the dataset by oversampling patches proportionally from the under-represented cities. We also used a city-stratified, randomly selected 80/10/10 split for our training/validation/test sets.

The model output resolution is limited by the spatial resolution of the target data we have for validating the model. LUR models have the finest spatial resolution for this purpose. The aim of this study is to leverage the meter-scale land surface information to map pollution. However, instead of pixel-to-pixel mapping, we used a broader land surface area that will allow us to accurately understand the pollution distribution in the area. To achieve this, we designed a patch sampling to collect a 40x40-pixel image centered over the LUR pollution point (Fig.1). The input to the model is a single patch of satellite imagery i.e. 40x40 pixels of 2.5m spatial resolution imagery which corresponds to a real world image patch of 100m x 100m in size (for PM2.5; NO2 patches are twice as big). Each patch corresponds to one LUR value from a continuous LUR surface. The model is learning to engineer features from the imagery and associate those features with the corresponding AQ measurement.

The patch dataset of imagery for London, LA, and Vancouver is split into 3 portions. 80% of the patches are used to train the model, 10% of the patches are used to examine performance between models, adjust model hyperparameters, and evaluate overfitting. The last 10% is held out until the end and is only used to evaluate performance of the final model. All numbers in the results section use the latter.

#### Validation

Stage 1: We used the trained model to predict on New York City (NYC) - a location completely unseen by the model during training - to examine generalizability. Stage 2: We examined the association between the model predictions and ground monitoring site concentrations in all study regions.

## 3 Results

### Air Quality Data

All studied regions are urban environments that have available coverage of satellite imagery, ground monitoring sites, and LUR-modeled target data at high spatial resolution. LUR models require substantial time and effort, mainly in obtaining a large number of input datasets, and while they are used for planning and decision-making purposes, LUR-modeled data is not commonly available.

**Fig. 2:**
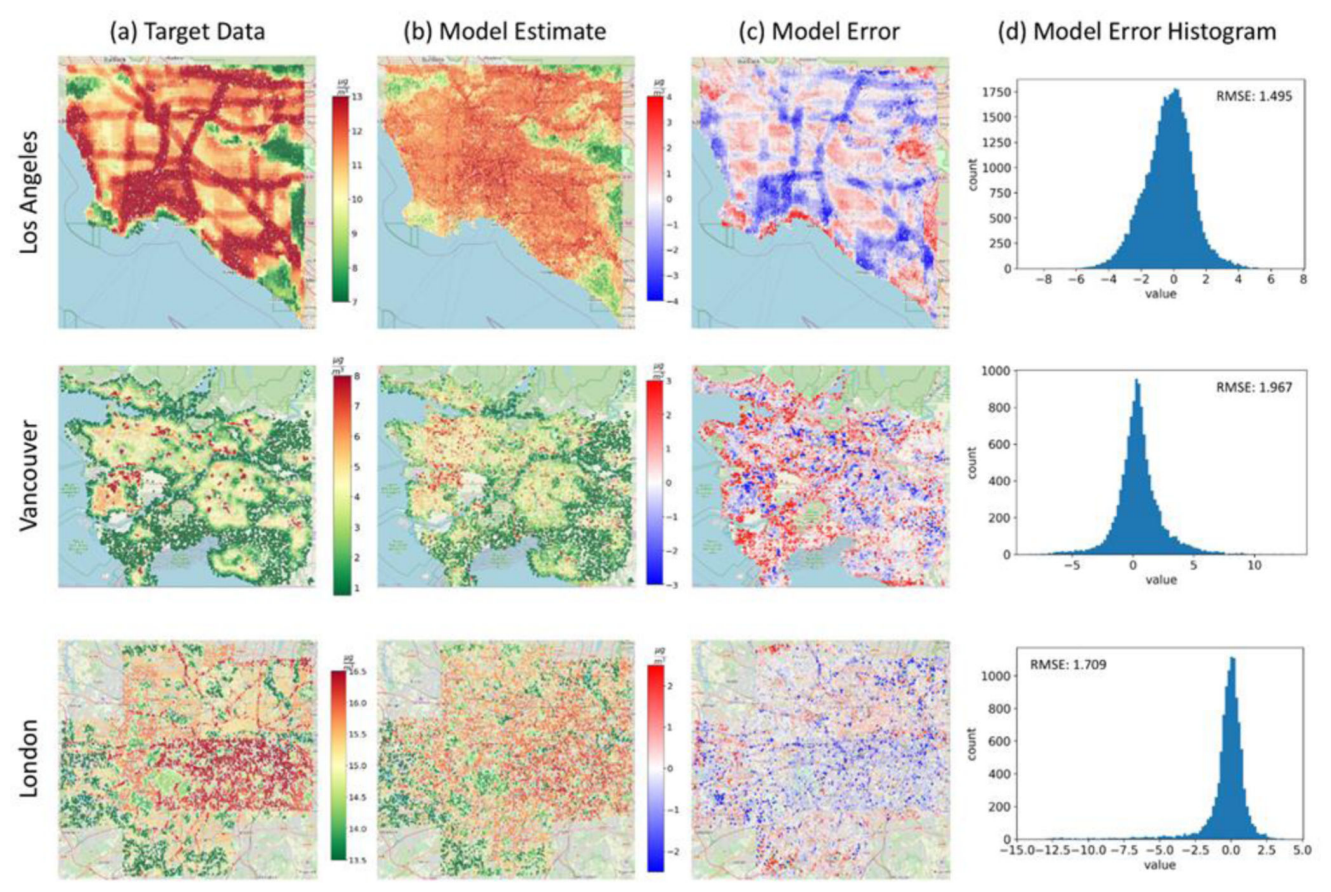
PM_2.5_ target data, model estimates and error PM_2.5_ target data **(a)**, based on 2010 LUR data in Los Angeles, Vancouver, and London. Column **(b)** shows the 100 m model estimates of PM_2.5_ annual concentrations using the testing dataset (10% of the available data for all 3 cities), and column **(c)** shows the model residuals calculated by the difference between the predicted concentrations and the target data. The residuals’ distributions are shown in column **(d)**.

**Fig. 3:**
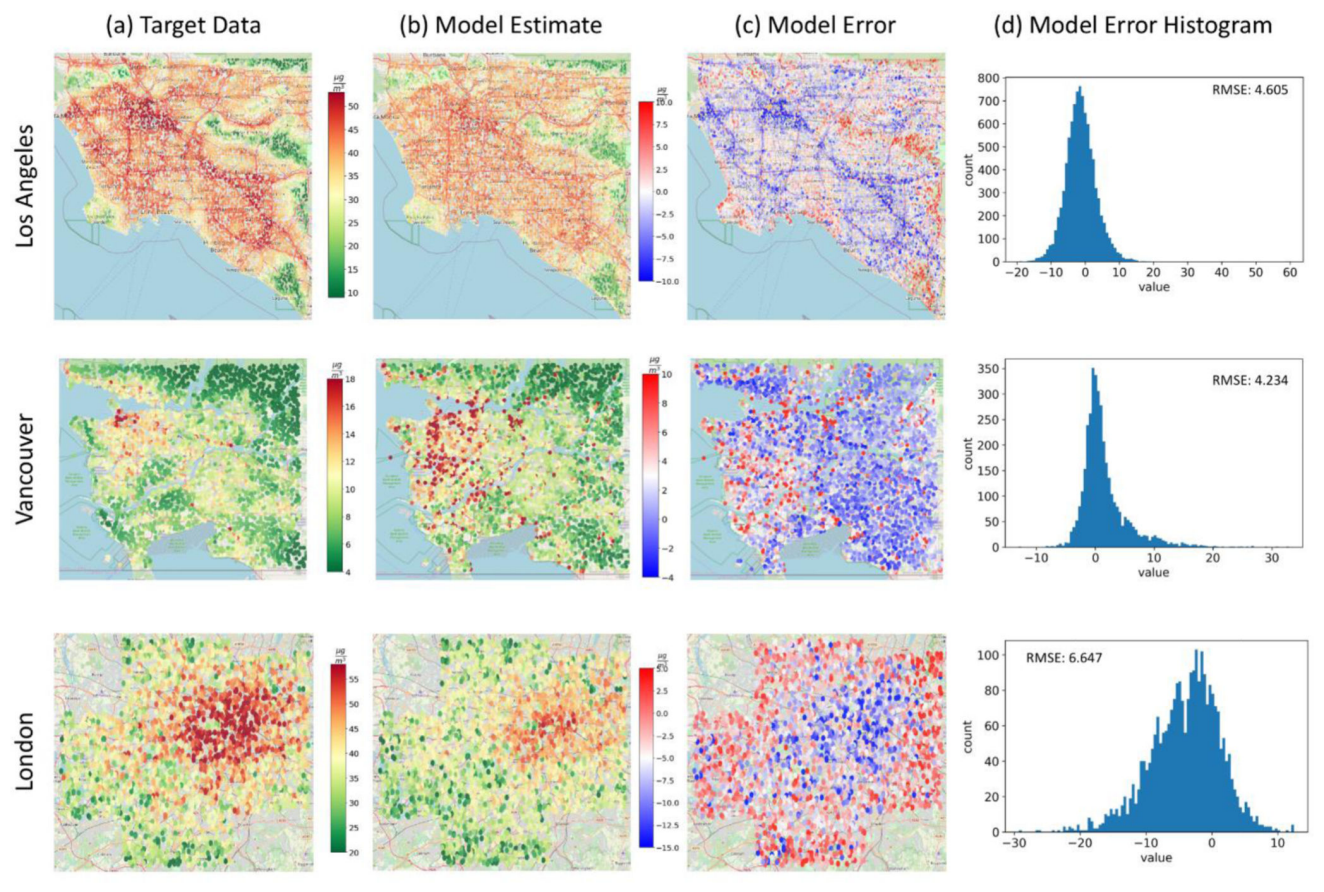
NO_2_ target data, model estimates and error NO_2_ target data **(a)**, based on 2010 LUR data in Los Angeles, Vancouver, and London. Column **(b)** shows the 200 m model estimates of NO_2_ annual concentrations using the testing dataset (10% of the available data for all cities). Column **(c)** shows the model residuals calculated by the difference between the predicted concentrations and the target data, and the residuals’ distributions are shown in column **(d)**.

The continuous LUR-modeled PM_2.5_ concentrations in all our case study regions are more heterogeneously spread, while NO_2_ concentrations demonstrate a clearer spatial trend, following roads as the main contributor (Fig. 2, Fig. 3). It is clear from the target data surfaces that PM_2.5_ annual concentrations are much lower in Vancouver than in LA and London, especially related to the city centers' density. (Details in Table 1).

In particular, the spatial distribution of PM_2.5_/NO_2_ in LA is dominated by highways and roads. Particularly high PM_2.5_/NO_2_ levels are observed in the downtown area and at the border of Los Angeles and Orange counties. Low pollution concentrations are spotted at recreation areas such as Angeles National Forest in Northeastern LA. Vancouver demonstrates lower PM_2.5_/NO_2_ concentrations compared to LA and is mainly dominated by recreational areas with low pollution levels. Downtown Vancouver and areas close to the Vancouver International Airport are regions with relatively higher pollution levels. The PM_2.5_/NO_2_ concentrations over London are mostly moderate with distinct high-pollution areas close to roads. Low pollution levels are observed in the southern and western regions co-located with South Downs National Park and North Wessex Downs Area of Outstanding Natural Beauty, respectively. NYC has a high concentration of pollutants in the densely populated Manhattan region, as well as Queens and Brooklyn Heights.

### Estimating PM_2.5_ Concentrations

The available satellite images were divided into 600,000 (100 m x 100 m) image patches from all study regions (details in the Methodology section) to train, validate, and test the PM_2.5_ estimation model (Table 2). The model was trained for 100 iterations and showed a Pearson correlation between the target data and the model-predicted PM_2.5_ concentrations in the testing subset of R=0.93, and an RMSE of 1.64 μ/m^3^, for all locations trained by the model. The model explained 87% of the variance in the multi-location testing dataset using solely images as input to the model, and the model outcomes for validation and testing phases result in a relatively low RMSE < 2 μ/m^3^ for all study regions (Fig. 2; Table 2).

The model captures most of the coarser spatial trends, like greenery spaces and populated areas, with fairly small residuals spanning mostly from -4 to +4 μg/m^3^. It also captures some of the spatial trend of larger road features in LA. However, it tends to underestimate pollution levels over roads (more dominant in LA) and overestimate over green areas (seen in all locations). All validation residuals are distributed fairly evenly around 0 μg/m^3^ for all locations (Fig. 2).

### Estimating NO_2_ Concentrations

Here we used the same imagery input from all study regions with an adjusted patch size of 200 m x 200 m, derived from the target data resolution, to estimate NO_2_ annual concentrations. We obtained results with a reasonably strong Pearson correlation between the predicted concentrations and target data (LUR) NO_2_ concentrations for all training locations of R=0.95, and a low RMSE μ 6.7 g/m^3^ (Fig. 3; Table 2). These results demonstrate a robust model. The model explained 91% of the variance in the data using solely the satellite imagery as input to the model. The model clearly captured patterns in the spatial distribution of NO_2_, which are largely affected by local sources, like traffic, as seen in the results (Fig. 3). However, the model underestimates highly polluted roads, and overestimates green areas with very low pollution-similar to the PM_2.5_ model results. Although the London city center is well captured by the model, its surrounding road networks are underestimated to a level of 15 μg/m^3^. This may reflect the greater proportion of diesel vehicles and higher levels of congestion in London compared with the other training cities (O’Driscoll *et al* 2018). LA and Vancouver show better results with more evenly distributed model residuals.

### Validation

**Fig. 4:**
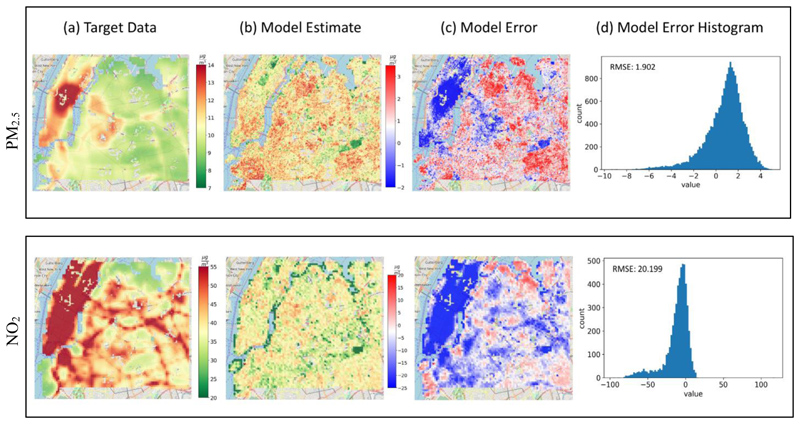
PM_2.5_ and NO_2_ target data, model estimates and error for NYC Target data **(a)**, based on 2010 LUR data in New York City. Column **(b)** shows the 100 m, and 200 m model estimates of PM_2.5_ and NO_2_ annual concentrations, respectively. Column **(c)** shows the model residuals calculated by the difference between the predicted concentrations and the target data, and the residuals’ distributions are shown in column **(d)**.

Stage 1: The model performed well (see Fig. 4 and Table 2) but lacks some of the feature-specificity the trained cities have.

The model clearly underestimates pollution over Central Park, which is an atypically large 'green lung' located in the middle of Manhattan—a bustling and densely populated borough of NYC. In the satellite images, these 'green lungs', which are large, green areas surrounded by high levels of human activity, look virtually indistinguishable from green areas outside the city, especially for small patch sizes.

**Fig. 5:**
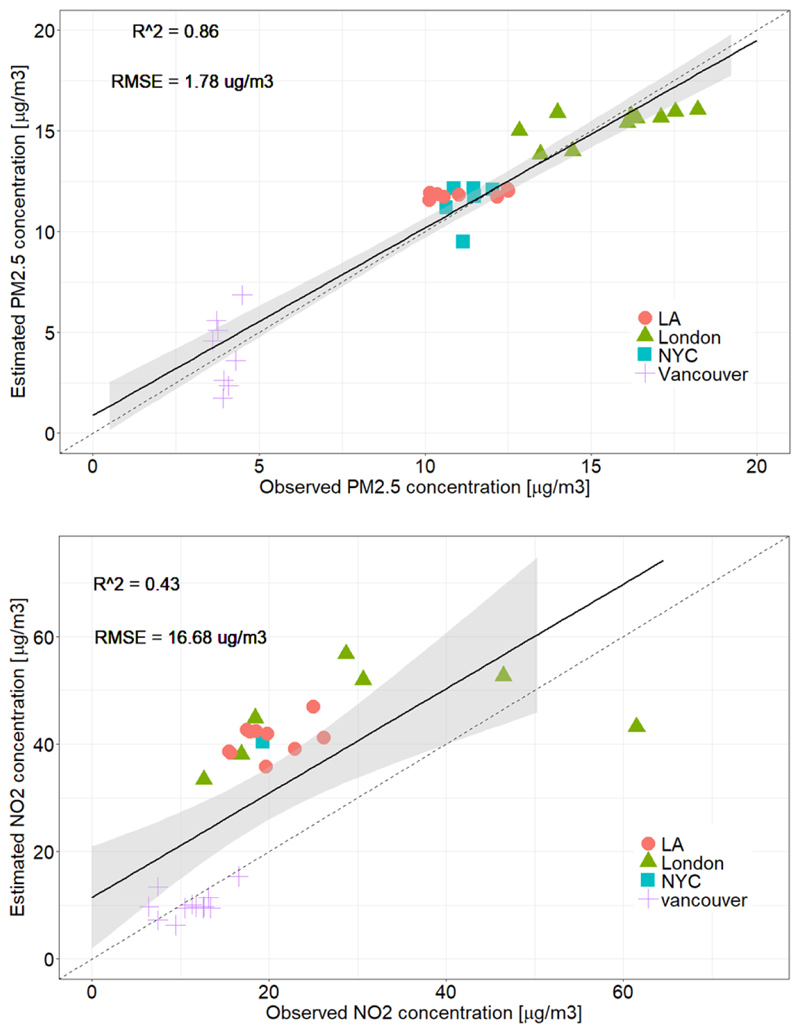
Correlations between observed and predicted PM_2.5_ and NO_2_ Correlation between observed PM_2.5_ **(upper panel)** and NO_2_ **(lower panel)** concentrations at ground monitoring sites and predicted values based on our model; the dashed line is the 1:1 line and the black line is the regression line with 95% confidence intervals (R^2^=0.86, 0.43, and RMSE=1.78, 16.68 μg/m^3^, respectively). Only sites that had co-located model-estimated values within a distance less than 500 m were included. For London, daily station data was used to calculate annual means of measured pollution over ground stations that had at least one measured value every month. For the other cities, annual means of measured data were used for the stations that had collected data for over 50% of the year.

Stage 2: Fig. 5 shows the correlation between the estimated annual PM_2.5_ and NO_2_ concentrations and the observed annual mean concentrations at co-located sites with a total R^2^ of 0.86, 0.43 and RMSE of 1.78, 16.68 μg/m^3^, respectively. We obtained data from 33 sites for PM_2.5_ and 31 sites for NO_2_.

### Model Interpretability

**Fig. 6:**
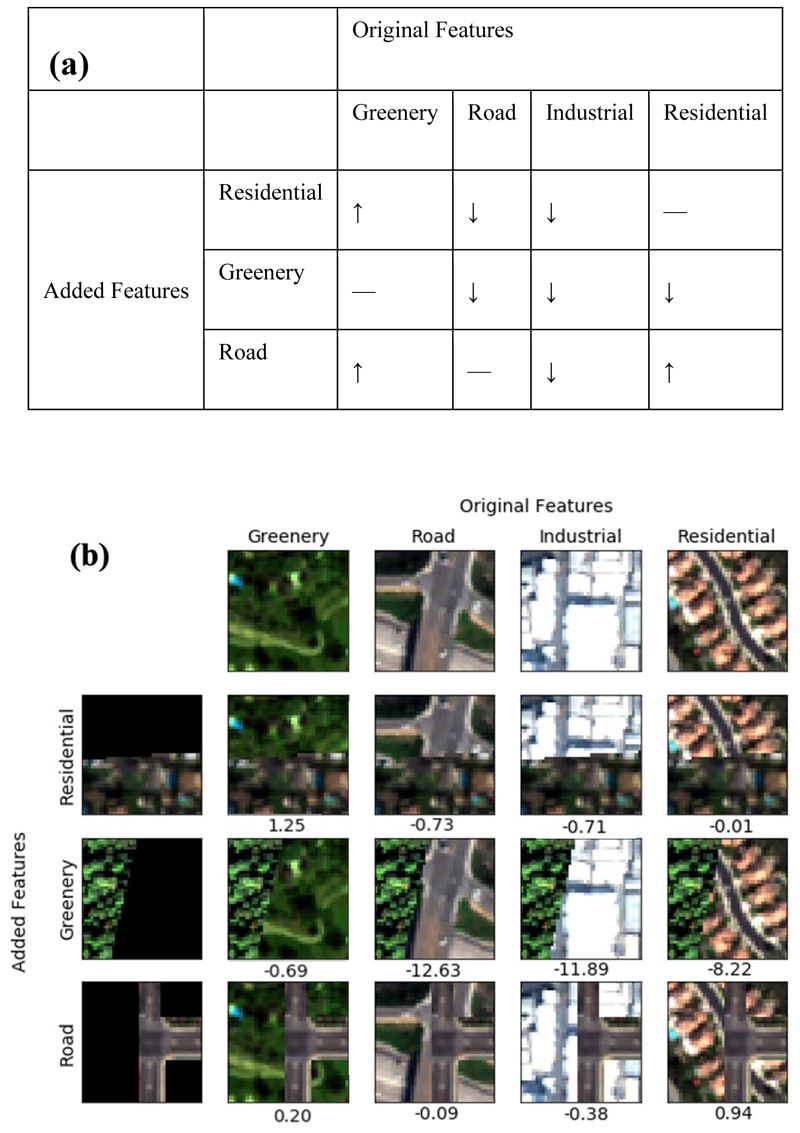
Feature contribution analysis (a) Hypothesis of expected changes in PM_2.5_ concentrations (μg/m^3^) when adding different urban features to original imagery. (b) Confusion table following the hypothesize in subfigure (a) using satellite images with and without synthesized residential, greenery, and road features. The columns represent the original satellite images of greenery, road, industrial, and residential from left to right. From top to bottom, the rows are partial residential, greenery and road images to be overlayed on original images. We demonstrate the differences of PM_2.5_ levels between the original and synthesized images on the right hand of each subplot. The results in subfigure (b) are in-line with intuition demonstrated in subfigure (a) and support our hypothesize that our model is learning the non-linear relation between urban objects and pollution concentrations.

To further examine the main hypothesis that urban features visible from high-spatial-resolution satellite images directly correlate with pollution levels, we analyze the effect of different urban features on the value of the prediction. We wanted to investigate whether inputs (e.g., roads, trees) to the LUR models (whose outputs were used as labels) are being captured by the deep learning models. Neural networks are known to often be black-box models with learned features that cannot be interpreted easily (Fong and Vedaldi 2017, Yosinski *et al* 2015, Simonyan *et al* 2013). However, recent advances in machine learning have made it possible to get insights into some types of neural networks, like CNNs. We know that certain urban features, such as roads and industrial objects, are correlated with high pollution levels. Conversely, green areas and residential blocks are associated with lower pollution levels (Guo *et al* 2019). To test the effect these urban features, have on the pollution estimate, we create synthesized images by adding urban features on top of existing imagery, and we use these synthesized images to examine how these added features affect the model estimate (Fig. 6b).

We started by randomly selecting different images that have urban features consisting of greenery, road, industrial and residential objects. Then we synthesized satellite images with urban features associated with low and high pollution levels (i.e., artificially adding image features over existing images) with examples in Fig. 6b Each row column in Fig. 6b represents an urban feature category and the intersection of each row and column shows a synthesized image combining those features. the differences between pollution concentrations of original and synthesized images predicted by the model are indicated on the right hand of each subplot in Fig. 6b We fed the original and synthesized images into the trained model and analyzed the difference in pollution levels predicted by the model.

In Fig. 6a we hypothesize the expected changes in PM_2.5_ concentrations between the original imagery and the synthesized imagery. For example, we expect that for all categories of features that added greenery features will lower the concentration. The correspondence between the expected changes (Fig. 6a) and the interpretability results (Fig. 6b) supports the hypothesis that our model learns the non-linear relations between visual urban features and their corresponding pollution levels. The overall results indicate the trained model successfully learns correlations between visual urban features and pollution levels in line with our physics-backed understanding of urban feature compositions.

## 4 Discussion

The goal of our study is to estimate hundred-meter-scale PM_2.5_ and NO_2_ concentrations in different urban environments. Our approach advances the science by (i) introducing a novel approach to the Earth Science community by applying deep learning to solely very high-spatial-resolution satellite imagery for AQ estimation, (ii) developing fine-scale continuous AQ maps, and (iii) creating an efficient approach for high-spatial-resolution AQ monitoring independent of ground monitoring and ancillary data. While we have compelling results, we recognize some of the limitations of our model, and discuss methods to address them.

The existing literature on satellite-based AQ estimation incorporates many variables in the modeling scheme, including meteorology, traffic, topography, and distance from emission source (Kloog *et al* 2015, van Donkelaar *et al* 2016, Hammer *et al* 2020). The model by Gupta et al. (2006) (Gupta *et al* 2006) reported 36% explained variation in PM_2.5_ in NYC, with a coarser (kilometer-scale) spatial resolution. Other models reported a PM_2.5_ prediction RMSE of 3.1 μg/m^3^ in the LA region (Wang *et al* 2016), and PM_2.5_ (NO_2_) hourly prediction RMSE of 6.7 (43.5) μg/m^3^ in London (Carslaw *et al* 2013). Our model results, trained and validated on modeled LUR data, improve current modeling efforts in all study areas (Table 3). The models exhibit reduced error and produce high-spatial-resolution predictions, while significantly decreasing the amount of input datasets required.

Certain features of the urban environment demonstrated a clear effect on the air quality prediction (e.g., greenery, roads). Nevertheless, changes in air pollution can be related to urban features that can't be recognized by the image-analysis model (e.g., the use of electric cars, winds, and temperature). These features may also affect the physical relationship between the measured air quality variables and the image reflectance values. Furthermore, the model underestimates PM_2.5_ and NO_2_ concentrations in certain urban areas that lack context - the aforementioned 'Central Park problem'. The model perceives Central Park only as a green area, without any context of the human activity in the areas surrounding it. This causes it to under-predict air pollution over that area. Incorporating "bigger picture" context into the model is expected to resolve this issue. We examined the use of satellite-based night-time light data retrieved from the Visible Infrared Imaging Radiometer Suite (VIIRS) instrument onboard the Suomi-NPP satellite (Stokes *et al* 2019) as a means for incorporating context-based on human activity into the model. The VIIRS night-time light product over NYC was analyzed as a potential additional input to the model. This dataset can detect the large, bright areas around Central Park that represent its location with context (i.e., in the heart of urban activity) (Fig. S2).

Apart from adding larger-scale context to the model, it was also observed that the VIIRS night-time light data demonstrated a high pixel-to-pixel Pearson Correlation Coefficient of 0.64 with PM_2.5_ over NYC. This shows promise for night-time light data to also be used as a feature co-located with the satellite images. Moreover, the VIIRS data is available globally, which could help facilitate the process of transferring the model to different urban regions around the globe. While we have demonstrated generalizability to a completely unseen city, we need to explore how well the model generalizes to a wide range of global urban environments in developed and developing locations with different distributions of both urban features and AQ. This study sets the floor for future work applying this model to other developed urban environments as well as examining the transferability of the model to developing cities that lack ground measurements. The ability to exclusively use satellite images to infer AQ on a local scale is novel to the AQ-modeling research community and can address some of the shortcomings of current modeling approaches, particularly in areas where historical ground data does not exist. This paper presents a strong and feasible methodology to work with for future AQ-modeling efforts. Although satellite images are a promising source of data for generating estimates at high-spatial-resolution on a local scale, as they do capture some spatial variability, they are limited to generalize in certain areas. Future work should focus on combining additional datasets readily available globally (including additional bands of satellite data) that can be combined with meter scale satellite images to generate better estimates.. In addition, future efforts will need to assess the sensitivity of image-based models to images collected with different temporal aspects such as time of day and season.

## Supplementary Material

Fig. S1, Fig. S2

## Figures and Tables

**Table 1:** Summary details of data availability over the study areas (2010)

No. of	London	Vancouver	Los Angeles	NYC
Available images (patches)	61 (105,242)	337 (117,924)	315 (369,602)	4 (19,480)
Annual mean LUR PM2.5/NO2 (µg/m^3^)	14.4/41.0	2.22/8.07	7.48/37.8	9.46/47.4
Co-located PM_2.5_/NO_2_ ground monitoring sites*	11/7	8/12	8/10	6/2
**Ground monitoring sites - PM2.5/NO2**				
Annual Mean (µg/m3)	16.17/61.32	5.65/8.11	10.91/25.99	10.17/-
Annual SD (µg/m3)	2.43/27.85	2.49/4.63	1.85/8.71	1.64/-

* Only sites that had co-located model-estimated values within a distance less than 500 m were included. For London, daily station data was used to calculate annual means of measured pollution over ground stations that had at least one measured value every month. For the other cities, annual means of measured data were used for the stations that had collected data for over 50% of the year.

**Table 2:** PM_2.5_ and NO_2_ model performance

City	PM_2.5_ Model			NO_2_ Model	
	RMSE (µg/m^3^)	NRMSE		RMSE (µg/m^3^)	NRMSE
Los Angeles (LA)	1.495	0.743		4.605	0.480
Vancouver	1.967	0.592		4.234	0.987
London	1.709	1.192		6.647	0.551
New York City (NYC)	1.902	1.499		20.199	1.776
Combined (just training cities)	1.64	0.321		4.925	0.165
**Combined (all cities)**	**1.706**	**0.484**		**11.107**	**0.682**

**Table 3:** Baseline model performance

Study	Variable	Study Region	R^2^	RMSE [μg/m^3^]
Gupta et al. 2006 ^30^	PM_2.5_	NYC	0.36	Not reported
Wang et al. 2016 ^31^	PM_2.5_	LA	0.80	3.10
Carslaw et al. 2013 ^32^	PM_2.5_	London	0.46	6.70
	NO_2_		0.50	43.50
**Current model**	PM_2.5_	All cities*	0.86	1.78
	NO_2_		0.43	16.68

* Calculation performed between model estimates and ground monitoring sites.

## Data Availability

Publicly available datasets, code, and maps resulting from this project will be archived for free download at NASA Ames Research Center (ARC) on the NASA Earth Exchange (NEX) platform website/data repository, and NASA GitHub, uploaded and archived in NASA PubSpace. Manuscripts will be deposited to PubSpace within one year of completion of the (manuscript) peer review process, according to NASA requirements. Commercial Satellite images are not publicly available and cannot be shared.
