# Supplementary material for: A Deep Learning Approach for Meter-Scale Air Quality Estimation in Urban Environments Using Very High-Spatial-Resolution Satellite Imagery
Source: Atmosphere (Basel). Author manuscript; Available in PMC 2023 Sep 18. (PMC7615102; doi:10.3390/atmos13050696)
Supplement: Fig. S1, Fig. S2 [file EMS187697-supplement-Fig__S1__Fig__S2.pdf]

A Deep Learning Approach for Meter-Scale Air Quality Estimation in Urban Environments Using Very High-Spatial-Resolution Satellite Imagery

Supplementary Information

Meytar Sorek-Hamer[a,b,*], Michael von Pohle[a,b], Adwait Sahasrabhojanee[a,b], Ata Akbari Asanjan[a,b], Emily Deardorff[a,b], Esra Suel[c], Violet Lingenfelter[a,b], Kamalika Das[a,b], Nikunj Oza[b], Majid Ezzati[c], and Michael Brauer[d]

[a] Universities Space Research Association (USRA), Mountain View, CA;
[b] NASA Ames Research Center, Mountain View, CA;
[c] Imperial College, London, UK;
[d] University of British Columbia, SPPH, Vancouver, Canada

*msorekhamer@usra.edu

A. Deep Learning Model Architecture

The implemented deep learning model (introduced in Methodology section) contains 3 blocks of 2 consecutive convolutional layers followed by a MaxPooling layer. The 3 blocks are followed by block of 3 convolutional layers and batch normalization layer. Finally, a series of data flattening, dense layer, batch normalization, and two dense layers will route the feature data into a continuous $PM_{2.5}$ and NO_2 levels (Fig S1). Adapted from VGG16, we used ReLU activations for all the convolutional and dense layers except the last dense layer. The chosen optimizer for training was Adam[43] with 0.001 and 0.9 learning rate and momentum, respectively. Sufficient number of epochs have been used for training the model and we ensured that the model converges without overfitting. The model was implemented in Python using Keras (https://keras.io/) and Tensorflow (https://tensorflow.org/) libraries.

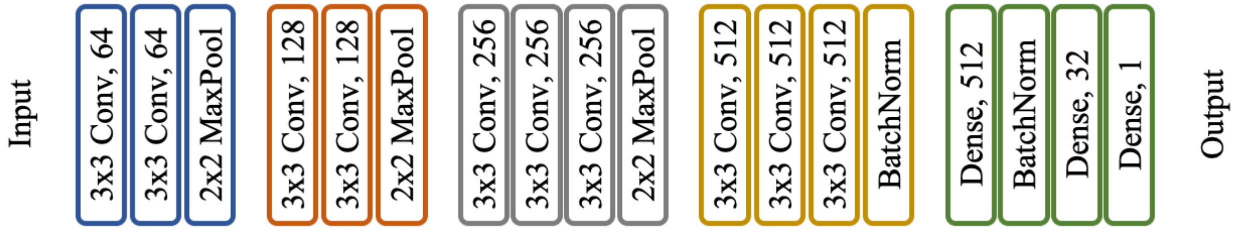

Fig. S1. Deep Learning Model Architecture The proposed model used for estimating $PM_{2.5}$ and NO_2. The model is a modified version of VGG16 and contains 4 blocks of convolutional layers (color-coded from top block to the fourth block), and a block of dense layers following the convolutional blocks to estimate the air quality values. For convolutional layers, the kernel sizes are indicated first, following by the number of features. The reported numbers for Max Pooling and dense layers are the pool size and number of hidden nodes, respectively. Inputs of this model are the visual bands from WorldView satellites at 100 m and 200 m resolutions for $PM_{2.5}$ and NO_2, respectively. The outputs are the corresponding values for $PM_{2.5}$ and NO_2.

B. Variables Used in the LUR Models

The following table lists the major variables used to calculate LUR models [1]:

Table S1. Common variables used in LUR models

(1) Air pollution monitoring data

- ESCAPE annual mean concentrations for 2009-2010 for NO_2 and $PM_{2.5}$.
- Annual mean concentrations for $PM_{2.5}$ and NO_2 for 2010 were also derived from the AIRBASE v8 dataset.

(2) Satellite derived air pollution estimates

- Satellite derived (SAT) estimates of $PM_{2.5}$ extracted from the global datasets reported in [2] at 10km resolution.
- For NO_2, SAT estimates were obtained from the tropospheric NO_2 columns measured with the OMI (Ozone Monitoring Instrument) on board the Aura satellite and were related to ground-level concentrations using global GEOS-Chem model, producing an annual gridded NO_2 surface for the year 2010 at a 10km resolution.

(3) Chemical transport model estimates

- Long range chemical transport model (CTM) estimates for $PM_{2.5}$ and NO_2 were derived from the MACC-II ENSEMBLE model, for the year 2010 at 0.1° x 0.1° (~10km) resolution.

(4) GIS predictor variables

- A spatial moving window summation function (focalsum in ArcGIS10) was used to calculate the local predictor variables (e.g. length of road and areas of different land covers) for selected distances.
    - Road data originated from the 1:10,000 EuroStreets digital road network (version 3.1, based on TeleAtlas MultiNet TM for year-2008).
    - Corine Land Use
    - Elevation data from SRTM Digital Elevation Database version 4.1.

C. VIIRS Night-Time Light Imagery

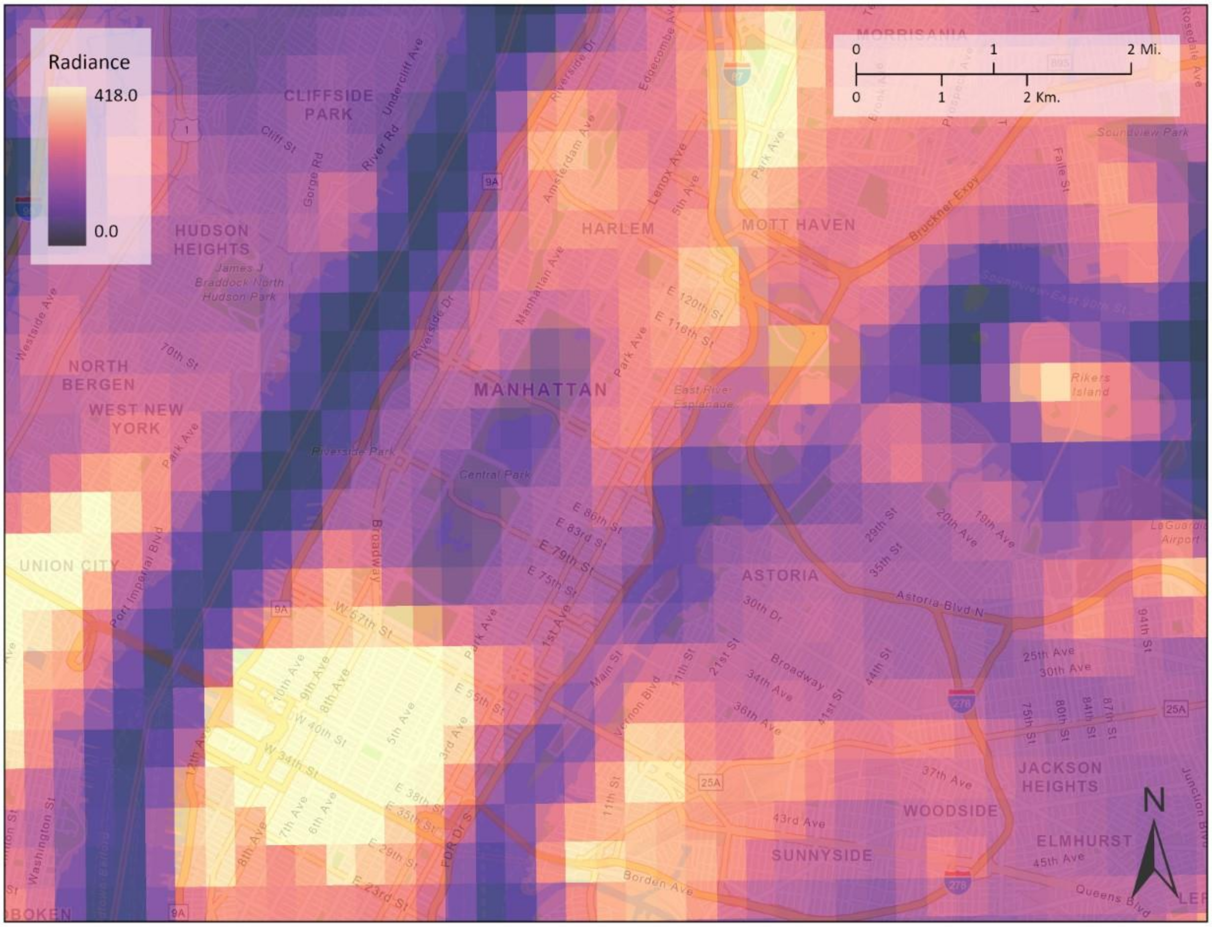

Fig. S2 VIIRS Night-Time Light over Manhattan. Night-time light data highlights areas of high urban activity, like the ones around Central Park (Reproduced from [3]). © OpenStreetMap contributors).

References

1. de Hoogh K, Gulliver J, Donkelaar A van, Martin R V, Marshall J D, Bechle M J, Cesaroni G, Pradas M C, Dedele A, Eeftens M, Forsberg B, Galassi C, Heinrich J, Hoffmann B, Jacquemin B, Katsouyanni K, Korek M, Künzli N, Lindley S J, Lepeule J, Meleux F, de Nazelle A, Nieuwenhuijsen M, Nystad W, Raaschou-Nielsen O, Peters A, Peuch V-H, Rouil L, Udvardy O, Slama R, Stempfelet M, Stephanou E G, Tsai M Y, Yli-Tuomi T, Weinmayr G, Brunekreef B, Vienneau D and Hoek G 2016 Development of West-European PM2.5 and NO2 land use regression models incorporating satellite-derived and chemical transport modelling data. *Environ. Res.* **151** 1–10
2. van Donkelaar A, Martin RV, Brauer M, Boys BL 2015 Use of satellite observations for long-term exposure assessment of global concentrations of fine particulate matter. *Environ Health Perspect* **123**:135–143;
3. ESRI (2018), Open Street Map (OSM), https://www.esri.com/arcgis-blog/products/arcgis-living-atlas/mapping/new-osm-vector-basemap/, Accessed: 7/1/2021
